# Association between non-high-density lipoprotein cholesterol to high-density lipoprotein cholesterol ratio and serum thyroid function measures: Recent Findings from NHANES 2007–2012 and Mendelian randomization

**DOI:** 10.3389/fendo.2025.1467254

**Published:** 2025-01-24

**Authors:** Mo-Yao Tan, Ping Zhang, Shan Wu, Si-Xuan Zhu, Ming Gao

**Affiliations:** ^1^ Chengdu Integrated TCM and Western Medicine Hospital, Chengdu, Sichuan, China; ^2^ Chengdu University of Traditional Chinese Medicine, Chengdu, Sichuan, China

**Keywords:** NHHR, serum thyroid function, NHANES, mendelian randomization, clinical practice

## Abstract

**Objective:**

There is limited epidemiological data regarding the association of blood lipids with thyroid hormones. Thus, the present article aims to explore whether there is an association between non-high-density to high-density lipoprotein cholesterol ratio (NHHR) and thyroid hormones.

**Methods:**

We analyzed samples from 3,881 adults aged 20 years and above who took part in the National Health and Nutrition Examination Survey (NHANES) spanning 2007 to 2012. The study tested for thyroid hormones, including total triiodothyronine (TT3), free triiodothyronine (FT3), total thyroxine (TT4), free thyroxine (FT4), as well as thyroid-stimulating hormone (TSH). Survey-weighted linear regression and restricted cubic spline (RCS) models were employed to investigate the relationship between NHHR and thyroid hormones. Subsequently, subgroup analyses were conducted. In Mendelian randomization (MR), the inverse variance weighting method (IVW) is used as the primary analytical approach.

**Results:**

This study finally comprised 3,881 adults aged 20 years and older. After extensive adjustments for covariables, the regression analysis revealed significant negative associations between NHHR and FT4 (β: -0.11, 95% confidence interval [CI]: -0.18, -0.04), FT4/FT3 (β: -0.06, 95% CI: -0.08, -0.04), and TT4/TT3 (β: -0.001, 95% CI: -0.001, 0.000). Both observational and Mendelian randomization studies suggest that high-density lipoprotein cholesterol, low-density lipoprotein cholesterol, and total cholesterol may not significantly influence the risk of hyperthyroidism or hypothyroidism.

**Conclusions:**

The study indicates negative associations between NHHR and FT4, as well as the ratios of FT4/FT3 and TT4/TT3. This suggests that NHHR may reflect changes in thyroid function, highlighting its potential clinical significance in assessing thyroid function and metabolic health.

## Introduction

Thyroid hormones consist of thyroxine (T4, approximately 80%) and triiodothyronine (T3, approximately 20%) (1). T4 is primarily released by the thyroid gland, whereas only about 20% of T3 is directly produced by the thyroid gland (2). The remaining 80% of T3 is converted from T4 in peripheral tissues by deiodinases (DIO1, DIO2) (2). Among these hormones, T3 is the active form, while T4 primarily serves as a precursor that can be converted into the active form (3). In the bloodstream, most T3 and T4 molecules are bound to serum binding proteins, with only a small fraction circulating freely as free thyroxine (FT4, approximately 0.03%) and free triiodothyronine (FT3, approximately 0.5%) (4). These free and bound portions together constitute total triiodothyronine (TT3) and total thyroxine (TT4) (5). The production of thyroid hormones is controlled by a complex negative feedback system involving thyroid-stimulating hormone (TSH) released by the pituitary gland (3). TSH regulates the synthesis and secretion of T3 and T4, while T3 and T4, in turn, regulate the production of TSH through negative feedback (3). T3 and T4 play crucial roles in many tissues, maintaining normal growth and metabolism, regulating energy expenditure and lipid balance, and playing key roles in the cardiovascular system (6). Studies have shown that abnormal levels of thyroid hormones, whether too high or too low, can have adverse effects on the body (7–9). Low levels of thyroid hormones can lead to coronary heart disease (7, 8), while high levels of thyroid hormones can lead to heart failure (9).

Some research has explored the association between thyroid hormones and lipid metabolism. For instance, research by Kim et al. on the Chinese population identified a significant positive association between FT4 and the levels of both high-density lipoprotein cholesterol (HDL-C) and low-density lipoprotein cholesterol (LDL-C) (10). Conversely, a study involving 2,183 adults found a negative association between FT4 and HDL-C (11). Similarly, Wang et al. reported a negative association between thyroid hormones and hyperlipidemia indicators, such as LDL-C (12). Non-high-density lipoprotein cholesterol (non-HDL-C) represents all cholesterol carried by lipoproteins other than HDL-C, including the cholesterol content in very low-density lipoprotein, low-density lipoproteins, and other lipoprotein particles (13). Furthermore, some research has shown that higher levels of non-HDL-C are linked to mild inflammation, which may facilitate the conversion of TT4 to TT3, thereby elevating TT3 levels (14, 15). However, one study suggested that activating inflammatory mediators and exposure to low temperatures could reduce FT3 levels (16).

Currently, the methods for measuring TT3 and TT4 are primarily immunoassays, but these methods have significant errors and limitations (17). A study questioned the accuracy of immunoassay results for low, normal, and high concentrations of TT3 and validated the results using the gold standard liquid chromatography-tandem mass spectrometry (LC-MS/MS) (18). The results showed that the measurements of TT3 using low-concentration immunoassay methods were not ideal (18). Additionally, a recent study by the IFCC C-STFT used various immunoassay methods to measure TT4 and TT3 in the serum of healthy individuals and compared the results with those reported by isotope dilution tandem mass spectrometry (ID-LC-MS/MS) (19). The findings revealed that most methods did not meet the optimal 5% target set by C-STFT, with only a few TT4 measurements falling within 10% of the reference values (19, 20). Particularly, since the concentration of TT3 is ten times lower than that of TT4, its measurement requires higher sensitivity and precision (21). However, most TT3 measurements exhibited a positive bias, indicating that the detection of TT3 and TT4 needs to be re-standardized (19, 20). Although tandem mass spectrometry provides higher accuracy in measuring TT3 and TT4, its complex operational steps, which require the separation of analytes from binding proteins to measure free thyroid hormones, make it difficult to be widely applied in clinical settings in the short term (22). The assessment of thyroid function primarily relies on thyroid hormones and TSH levels (23). Therefore, it is particularly important to establish new blood lipid indicators that can more accurately reflect fluctuations in these levels for evaluating thyroid function.

The non-high-density lipoprotein cholesterol to high-density lipoprotein cholesterol ratio (NHHR) is increasingly regarded as a comprehensive and innovative parameter for assessing lipid components in atherosclerosis (24). NHHR is associated with atherosclerosis and provides significant predictive value for various diseases. For instance, a study on the Asian population demonstrated an association between NHHR and non-alcoholic fatty liver disease; a one-unit increase in NHHR could lead to a 64.5% increase in the ratio of alanine aminotransferase to aspartate aminotransferase (25, 26). Since the liver is the primary site for converting TT4 to TT3 (27), non-alcoholic fatty liver disease indirectly affects thyroid hormone levels by altering the metabolic rate of liver function (28). Another study indicated that NHHR offers more accurate predictions for metabolic syndrome than traditional lipid markers (29). Metabolic syndrome is characterized by elevated lipid levels, which can disrupt the iodination process of TT4, potentially resulting in increased levels of FT3 and TT3 and a decrease in FT4 (30, 31). Therefore, exploring the association between NHHR and thyroid hormones and functions is scientifically significant and positions NHHR as a novel biomarker, paving new pathways for monitoring and regulating thyroid hormone levels.

Mendelian randomization (MR) analysis uses genetic variation, such as single nucleotide polymorphisms (SNPs), as instrumental variables (32). By exploiting the random allocation of genetic variation, MR excludes the influence of confounding factors and helps identify causal relationships (32). Therefore, the MR method can effectively assess the causal association between NHHR and thyroid hormones.

Based on the background information and prior research, we hypothesize that NHHR, as an innovative biomarker, more accurately reflects fluctuations in thyroid hormone levels. Therefore, our study investigates the association between NHHR and thyroid hormones in American adults using data from the NHANES survey conducted between 2007 and 2012. Additionally, the use of two-sample MR deepens the understanding of the causal relationship between these variables.

## Methods

### Study population

This study utilizes data from the NHANES, a cross-sectional survey conducted every two years by the Centers for Disease Control and Prevention (CDC). This program seeks to evaluate the nutritional and health status of residents in the U.S. The survey was performed in accordance with the relevant guidelines and regulations. The survey includes face-to-face interviews and physical examinations, encompassing demographics, socioeconomic status, and health-related information. The research obtained official endorsement from the Research Ethics Review Board in the National Center for Health Statistics, and every individual has signed the corresponding written informed consent (33).

**Figure 1:**
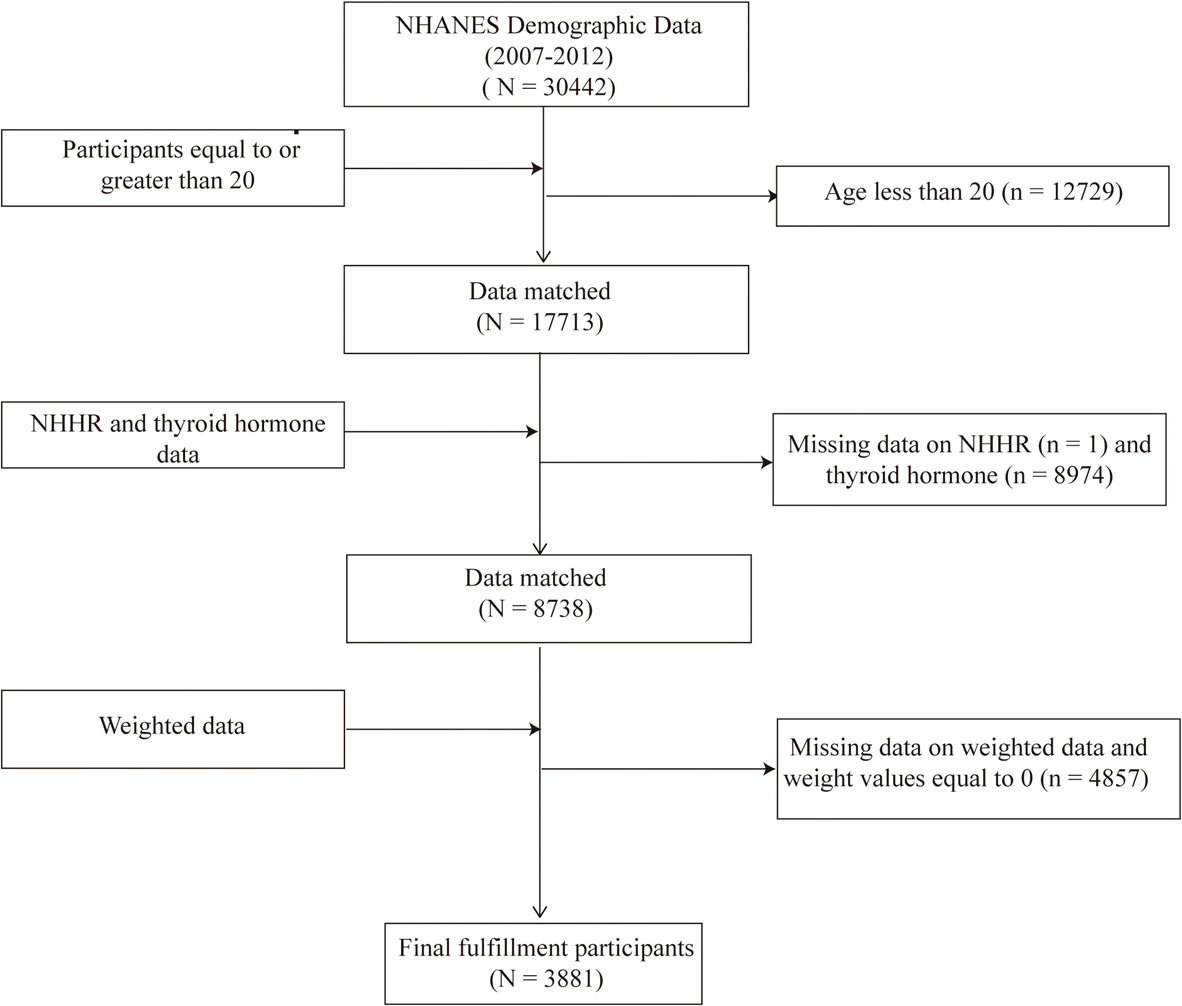
Flowchart of the sample selection from the 2007–2012 National Health and Nutrition Examination Survey (NHANES).

This study utilized data from three consecutive NHANES cycles (2007-2008, 2009-2010, and 2011-2012) because thyroid hormone data were only available during these specific periods. Initially, there were 30,442 participants. However, after excluding individuals under 20 years old (n = 12,729), those without thyroid hormone data (n = 8,974), those missing NHHR data (n = 1), and those with a weight of 0 or missing values (n = 4,857), the final sample size consisted of 3,881 participants. This final sample included individuals both with and without thyroid disorders such as thyroid cancer, hyperthyroidism, and hypothyroidism (**Figure 1**).

### Definition of NHHR

The NHHR was regarded as the exposure variable. The formula used was: NHHR = Non-HDL-C/HDL-C, where Non-HDL-C is calculated as total cholesterol (TC) minus HDL-C. To enhance the normality of the variables, we applied a log transformation on independent variables. The log2-transformed values closely approximate a normal distribution and exhibit homogeneity of variance (Levene’s test *P* > 0.05).

### Thyroid function assessment

This study considered thyroid function parameters such as FT3, TT3, FT4, TT4, and TSH as outcome variables, with data sourced from the NHANES laboratory data. The survey utilized competitive binding immunoassay to measure TT3, FT3, and TT4 and employed a two-step enzyme immunoassay for assessing FT4. TSH levels were measured using the third-generation bi-site immunoassay for access to ultra-sensitive human Thyroid-Stimulating Hormone (TSH) (34, 35). Furthermore, our research determined the TT4/TT3 and FT4/FT3 ratios to examine peripheral TT4 metabolic activity and also assessed the FT4/TT4 ratio to investigate how thyroid hormones interact with their binding proteins.

### Covariables

Drawing on prior research (36, 37), this study included multiple factors that could affect the relationship between NHHR and thyroid hormones. These factors include age, gender, race, marital status, urine iodine concentration, moderate recreational activities, education level, poverty-to-income ratio (PIR), body mass index (BMI), smoking status, alcohol consumption, diabetes, hypertension, lipid-lowering medications and thyroid hormone medications. Detailed information on all covariables can be found in **Supplementary Material 1**.

### Statistical analysis

Sampling weights, stratification, and clustering factors from the NHANES study were integrated into each statistical analysis to mirror the complex multi-stage sampling methodology precisely. This methodology guaranteed accurate estimation results and mitigated the risk of overstating statistical significance. When selecting weights, the NHANES official guidelines recommend first determining the smallest population group. In this study, we chose the specific sub-weight WTSAF2YR. For the combined survey cycles, according to the NHANES analysis guidelines, a new sampling weight is created by dividing the two-year weight of each cycle by three, resulting in a new weight. Continuous variables are reported as means ± standard errors and tested by one-way analysis of variance (ANOVA), while categorical variables are expressed as frequencies with the corresponding survey-weight percentages and assessed by weighted chi-square tests. Restricted cubic spline (RCS) analysis with three knots was employed to explore potential nonlinear relationships of NHHR with thyroid hormones. The knots were set at the 10th, 50th, and 90th percentiles of the NHHR. If the results demonstrated linear relationships, further linear regression analysis and subgroup analysis were conducted. The linear regression models were utilized to investigate the relationships between NHHR and various thyroid hormones. The coefficient (β), along with its 95% confidence intervals (CIs), were estimated. Meanwhile, logistic regression models were used to investigate the associations between HDL-C, LDL-C, TC, and hyperthyroidism and hypothyroidism. No covariables was adjusted in Model 1, and Model 2 incorporated adjustments for age, gender, and race. Model 3 extended these adjustments to encompass an array of variables, including age, gender, race, marital status, urine iodine concentration, moderate recreational activities, education level, PIR, BMI, smoking status, alcohol consumption, diabetes, hypertension, lipid-lowering medications and thyroid hormone medications. Additionally, a series of subgroup analyses were followed to investigate differences within specific populations. The subgroup factors considered include age (<60, ≥60), gender (male, female), smoking status (never, former, now), moderate recreational activities (inactive, active), stroke (no, yes), diabetes (no, yes), and hypertension (no, yes). Furthermore, we conducted sensitivity analyses to validate the stability of the results. We excluded participants with thyroid cancer, hyperthyroidism, and hypothyroidism to ensure the validity of the relationship between NHHR and thyroid hormones. Finally, the two-sample MR analysis was performed to evaluate the causal links between lipoprotein cholesterol and thyroid function. For this, we utilized GWAS data from the Global Lipids Genetics Consortium for HDL, LDL, and TC as exposures (38), and the UKB-based GWAS for hyperthyroidism and hypothyroidism as binary outcomes [from the Ben Elsworth study]. Genetic data for hyperthyroidism and hypothyroidism can be accessed via the following links: https://gwas.mrcieu.ac.uk/datasets/ukb-b-20289/ and https://gwas.mrcieu.ac.uk/datasets/ukb-b-19732/. We employed the multiplicative random-effects inverse-variance weighted (IVW) method, weighted median method, and MR-Egger regression for analysis. The Cochran’s Q test was used to test the heterogeneity among SNPs and the intercept from MR-Egger was used to assess the potential pleiotropy.

We used multiple imputation based on chained equations (MICE), implemented in the R MI package, to impute missing covariates with five iterations. Statistical significance was determined by a two-tailed *P* below 0.05. These analyses were conducted using the “TwoSampleMR” package (version 0.5.6) in R (version 4.1.2).

## Results

### Baseline characteristics of participants

**Table 1** shows that all included participants had a mean age of 46.93 ± 0.43 years, with males comprising 48.50% and females 51.50%. Notably, the thyroid hormone levels included an average TT3 of 115.37 ± 0.70 ng/dL, TT4 of 7.91 ± 0.05 µg/dL, FT3 of 3.23 ± 0.01 pg/mL, FT4 of 10.35 ± 0.07 pmol/L, and TSH of 2.14 ± 0.09 mIU/L. Additionally, significant differences were observed in BMI, TT3, and FT3 among participants in the fourth quartile group compared to those in lower NHHR quartiles (*P* < 0.05). Across different strata of NHHR, no significant differences were observed in age, TT4, TSH, urinary iodine concentration, PIR, marital status, hypertension, or CVD (all *P* > 0.05). The detailed information on the concentration of both independent and dependent variables can be found in **Supplementary Material 2**.

**Table 1:** Weighted baseline characteristics of participants.

NHHR	Total	Q1	Q2	Q3	Q4	*P*-value
**Age (years)**	46.93 (0.43)	46.46 (0.76)	47.98 (0.85)	46.78 (0.75)	46.51 (0.64)	0.43
**Body mass index (kg/m^2^)**	28.46 (0.12)	25.42 (0.22)	28.03 (0.24)	29.40 (0.23)	31.03 (0.25)	**< 0.001**
**TT3 (ng/dL)**	115.37 (0.70)	110.15 (1.24)	115.04 (1.35)	117.06 (1.08)	119.27 (1.26)	**< 0.001**
**TT4 (ug/dL)**	7.91 (0.05)	7.79 (0.08)	7.95 (0.08)	7.97 (0.08)	7.92 (0.05)	0.45
**FT3 (pg/mL)**	3.23 (0.01)	3.13 (0.02)	3.22 (0.02)	3.25 (0.02)	3.31 (0.02)	**< 0.001**
**FT4 (pmol/L)**	10.35 (0.07)	10.49 (0.13)	10.38 (0.10)	10.34 (0.09)	10.18 (0.10)	**0.04**
**TSH (mIU/I)**	2.14 (0.09)	1.99 (0.07)	2.08 (0.08)	2.08 (0.07)	2.41 (0.28)	0.50
**FT4/FT3**	3.25 (0.02)	3.39 (0.04)	3.27 (0.04)	3.22 (0.03)	3.11 (0.03)	**< 0.001**
**TT4/TT3**	0.07 (0.001)	0.07 (0.001)	0.07 (0.001)	0.07 (0.001)	0.07 (0.001)	**< 0.001**
**FT4/TT4**	1.34 (0.01)	1.38 (0.01)	1.34 (0.01)	1.32 (0.01)	1.31 (0.01)	**< 0.001**
**FT3/TT3**	0.03 (0.00)	0.03 (0.00)	0.03 (0.00)	0.03 (0.00)	0.03 (0.00)	**0.01**
**Urine iodine concentration (ug/L)**	252.79 (19.65)	300.02 (49.60)	229.66 (22.39)	194.54 (12.92)	290.53 (56.14)	0.06
**Sex, n (%)**						**< 0.001**
Male	1901 (48.50)	349 (33.20)	406 (40.65)	529 (55.00)	617 (65.00)	
Female	1980 (51.50)	624 (66.80)	560 (59.35)	441 (45.00)	355 (35.00)	
**Race, n (%)**						**< 0.001**
Non-Hispanic White	1792 (69.30)	455 (69.03)	442 (68.94)	447 (70.60)	448 (68.52)	
Non-Hispanic Black	732 (10.92)	243 (14.68)	206 (12.41)	156 (9.23)	127 (7.36)	
Mexican American	648 (8.22)	108 (5.64)	154 (8.03)	186 (8.95)	200 (10.28)	
Other Race	709 (11.56)	167 (10.65)	164 (10.62)	181 (11.22)	197 (13.84)	
**PIR, n (%)**						0.104
High-income	931 (35.41)	256 (36.78)	248 (37.67)	243 (35.88)	184 (31.27)	
Low-income	817 (14.46)	186 (13.36)	201 (14.15)	190 (12.53)	240 (17.97)	
Middle-income	2133 (50.13)	531 (49.86)	517 (48.18)	537 (51.59)	548 (50.76)	
**Recreational Activity, n (%)**						**0.002**
Inactive	2106 (46.69)	472 (41.76)	543 (45.34)	526 (47.01)	565 (52.77)	
Active	1775 (53.31)	501 (58.24)	423 (54.66)	444 (52.99)	407 (47.23)	
**Education Level, n (%)**						**<0.001**
High school or less	1591 (40.91)	337 (34.63)	369 (38.22)	417 (42.99)	465 (47.84)	
More than high school	2290 (59.09)	636 (65.37)	597 (61.78)	553 (57.01)	507 (52.16)	
**Marital Status, n (%)**						0.491
Married or living with partner	2381 (61.35)	551 (56.63)	594 (61.49)	618 (63.71)	619 (63.68)	
Widowed/Divorced/Separated/ Never married	1500 (38.65)	422 (43.37)	372 (38.51)	352 (36.29)	353 (36.32)	
**Smoking status, n (%)**						**0.009**
Never	2095 (55.11)	566 (58.30)	542 (56.10)	501 (51.65)	486 (50.00)	
Now	805 (20.61)	170 (16.74)	189 (19.57)	196 (20.20)	250 (25.72)	
Former	981 (24.28)	237 (24.96)	235 (24.33)	273 (28.15)	236 (24.28)	
**Alcohol intake, n (%)**						**0.004**
Never	565 (11.32)	152 (11.99)	154 (13.45)	140 (11.12)	119 (8.72)	
Mild	1227 (35.08)	309 (35.42)	316 (34.24)	309 (35.11)	293 (35.53)	
Moderate	537 (15.91)	161 (18.33)	141 (17.83)	113 (14.70)	122 (12.80)	
Heavy	821 (21.83)	208 (23.33)	182 (19.85)	213 (21.30)	218 (22.81)	
Former	731 (15.86)	143 (10.93)	173 (14.63)	195 (17.77)	220 (20.14)	
**Hypertension, n (%)**						0.08
No	2285 (64.89)	596 (68.50)	577 (65.55)	556 (64.00)	556 (61.44)	
Yes	1596 (35.11)	377 (31.50)	389 (34.45)	414 (36.00)	416 (38.56)	
**CVD, n (%)**						0.64
No	3040 (84.90)	850 (90.55)	863 (91.41)	869 (92.25)	861 (90.84)	
Yes	841 (15.10)	123 (9.45)	103 (8.59)	101 (7.75)	111 (9.16)	
**Diabetes, n (%)**						**0.005**
No	3040 (84.90)	784 (86.74)	782 (86.50)	767 (85.59)	707 (80.67)	
Yes	841 (15.10)	189 (13.26)	184 (13.50)	203 (14.41)	265 (19.33)	

All values are presented as number (n) and proportion (%) for categorical variables, assessed via weighted chi-square tests, or mean (standard error) for continuous variables, assessed via weighted Student’s t-tests.

Q1, Quartile 1; Q2, Quartile 2; Q3, Quartile 3; Q4, Quartile 4; PIR, Ratio of family income to poverty; CVD, cardiovascular disease; FT4, free thyroxine; TT4, total thyroxine; FT3, free triiodothyronine; TT3, total triiodothyronine; TSH, thyroid-stimulating hormone.

The bolded P-value indicates statistical significance.

### Nonlinear relationships between NHHR and thyroid hormones

**Figure 2:**
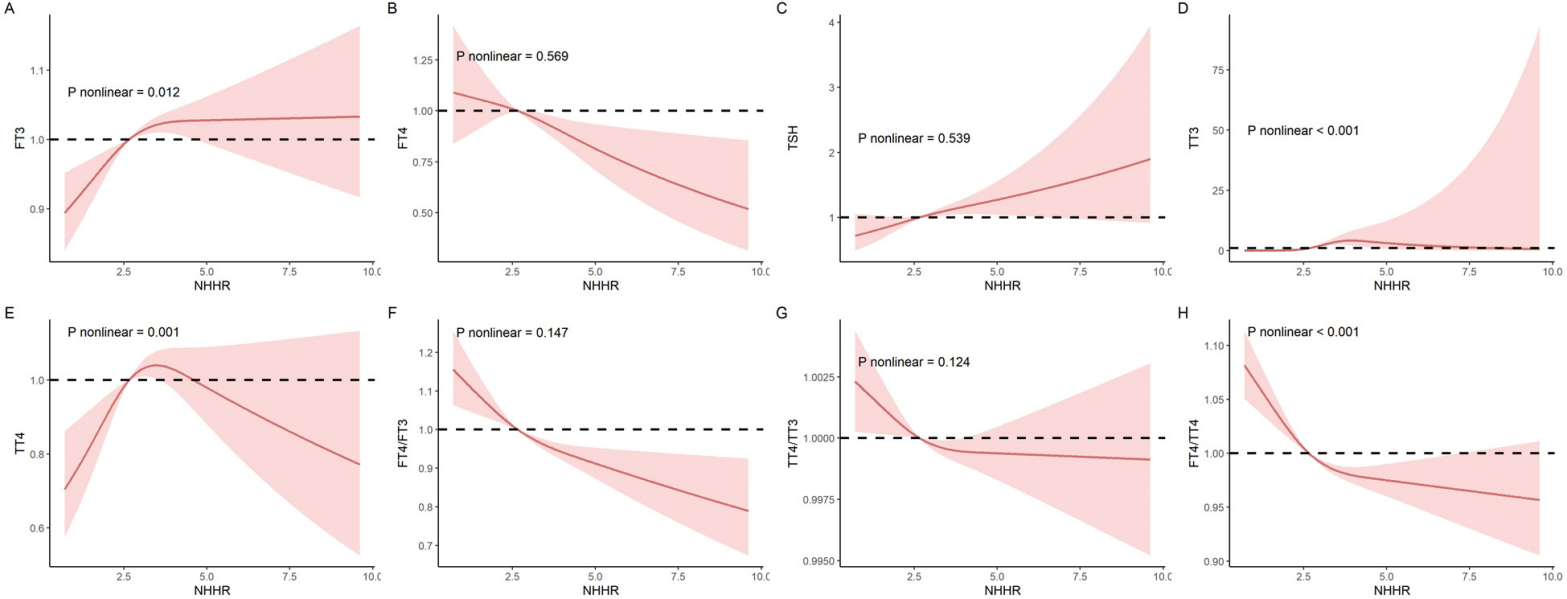
The nonlinear relationships between NHHR and thyroid hormones was investigated in the National Health and Nutrition Examination Survey (NHANES) conducted between 2007 and 2012. **(A)** FT3; **(B)** FT4; **(C)** TSH; **(D)** TT3; **(E)** TT4; **(F)** FT4/FT3; **(G)** FT4/FT3 **(H)** TT4/TT3; and **(I)** FT4/TT4.

Additionally, RCS models were applied to evaluate the nonlinear relationships between NHHR and thyroid hormones, as illustrated in **Figure 2**. In these RCS models, linear associations were noted between NHHR and FT4 (*P* for non-linearity trend: 0.569), TSH (*P* for non-linearity trend: 0.539), FT4/FT3 (*P* for non-linearity trend: 0.147), as well as TT4/TT3 (*P* for non-linearity trend: 0.124). Conversely, non-linear associations were distinctly observed with NHHR and FT3 (*P* for non-linearity trend: 0.012), TT3 (*P* for non-linearity trend < 0.001), TT4 (*P* for non-linearity trend = 0.001), and the FT4/TT4 ratio (*P-* value for non-linearity trend < 0.001).

### Association between NHHR and serum thyroid function indicators

**Table 2** displays the comprehensive results of linear regression using three different models evaluating the relationships of NHHR with thyroid function. The fully adjusted model revealed a negative association of NHHR with FT4 (β: -0.18, 95% CI: -0.32, -0.04), FT4/FT3 (β: -0.12, 95% CI: -0.16, -0.07), TT4/TT3 (β: -0.001, 95% CI: -0.001, 0.000), and FT4/TT4 (β: -0.12, 95% CI: -0.16, -0.07).

**Table 2:** Association between log2-transformed NHHR and serum thyroid hormones in U.S. adults.

serum thyroid hormones	β^a^ (95% CI), P-value		
	Model 1^b^	Model 2^c^	Model 3^d^
**FT4**	**-0.18 (-0.30, -0.05) 0.01**	**-0.20 (-0.33, -0.08) 0.002**	**-0.18 (-0.32, -0.04) 0.01**
**TSH**	0.19 (-0.08, 0.47) 0.17	0.28 (-0.10, 0.66) 0.14	0.29 (-0.13, 0.71) 0.16
**FT4/FT3**	**-0.16 (-0.20, -0.11) <0.001**	**-0.12 (-0.17, -0.08) <0.001**	**-0.12 (-0.16, -0.07) <0.001**
**TT4/TT3**	**-0.001 (-0.002, -0.001) <0.001**	**-0.001 (-0.001,0.000) 0.006**	**-0.001 (-0.001,0.000) 0.003**

Due to the non-linear relationships between NHHR and FT3, TT3, TT4, and FT4/TT4, their results are not displayed in the linear regression analysis;β^a^: effect size; Model 1^b^: no covariables were adjusted; Model 2^c^: adjusted for sex, age, and race; Model 3^d^: adjusted for age, gender, race, marital status, urine iodine concentration, moderate recreational activities, education level, PIR, BMI, smoking status, alcohol consumption, diabetes, hypertension, lipid-lowering medications and thyroid hormone medications

NHHR, non-high-density lipoprotein cholesterol to high-density lipoprotein cholesterol ratio; 95% CI, 95% confidence interval; OR, odds ratio; FT4, free thyroxine; TT4, total thyroxine; FT3, free triiodothyronine; TT3, total triiodothyronine; TSH, thyroid-stimulating hormone. The bold values indicate statistically significant differences.

### Association of HDL-C, LDL-C, and TC with hyperthyroidism and hypothyroidism

**Supplementary Material 3** presents the results of logistic regression analyses examining the associations of HDL-C, LDL-C, and TC with hyperthyroidism and hypothyroidism across three models. After adjusting for covariates, HDL-C (OR: 0.86, 95% CI: 0.38–1.93), LDL-C (OR: 0.81, 95% CI: 0.64–1.03), and TC (OR: 0.80, 95% CI: 0.65–1.00) showed no statistically significant associations with hyperthyroidism. Similarly, no significant associations were found between HDL-C (OR: 0.96, 95% CI: 0.84–1.10), LDL-C (OR: 0.98, 95% CI: 0.94–1.03), or TC (OR: 0.98, 95% CI: 0.93–1.03) and hypothyroidism.

Consistent with the cross-sectional findings from NHANES data, the MR analysis also revealed no significant associations between HDL-C, LDL-C, TC, and hyperthyroidism or hypothyroidism (*P* > 0.05). The detailed results are presented in **Table 3**. Furthermore, no evidence of horizontal pleiotropy was detected.

**Table 3:** MR results of causal links between lipoprotein cholesterol and thyroid function.

Exposure	Outcome	No.SNP	Methods	OR	95% CI	*P*	Horizontal pleiotropy *P*_for Egger intercept_	Heterogeneity *P*_for Cochran’s Q_
HDL	Hyperthyroidism	74	IVW	0.999	0.996, 1.002	0.719		1.515e-84
		74	Weighted median	1.000	0.998, 1.002	0.858		
		74	MR Egger	1.002	0.997, 1.008	0.448	0.260	
LDL		57	IVW	1.000	0.999, 1.001	0.751		1.345e-2
		57	Weighted median	1.000	0.998, 1.002	0.949		
		57	MR Egger	1.001	0.999, 1.003	0.585	0.383	
TC		70	IVW	1.000	0.998, 1.001	0.753		4.172e-4
		70	Weighted median	1.000	0.998, 1.002	0.962		
		70	MR Egger	1.001	0.998, 1.004	0.478	0.322	
HDL	Hypothyroidism	85	IVW	1.001	0.994, 1008	0.860		2.273e-100
		85	Weighted median	0.999	0.995, 1.003	0.522		
		85	MR Egger	1.002	0.989, 1.015	0.765	0.808	
LDL		77	IVW	0.999	0.994, 1.006	0.854		4.993e-113
		77	Weighted median	1.000	0.997, 1.003	0.848		
		77	MR Egger	1.003	0.994, 1.012	0.539	0.306	
TC		84	IVW	0.998	0.992, 1.005	0.635		2.080e-120
		84	Weighted median	0.999	0.995, 1.002	0.381		
		84	MR Egger	1.001	0.991, 1.012	0.799	0.484	

MR, Mendelian randomization; IVW, inverse-variance weighted; No.SNP, number of single-nucleotide polymorphism (SNP); SE, standard error; OR, odds ratio; CI, Confidence interval.

### Subgroup analysis

Subgroup analyses were conducted to explore the impact of specific population characteristics on the relationship between NHHR and thyroid hormones, as detailed in **Supplementary Materials 4**–**9**. Analyses by gender indicated negative associations between NHHR and FT4 and FT4/FT3 in both males and females. Notably, in females, a similar negative relationship was observed with the TT4/TT3 ratio. When analyzed by age, NHHR showed a consistent negative association with FT4/FT3 in both subgroups (≥60 and <60 years), with a positive relationship with TSH in the older subgroup and FT4 in the younger subgroup. Analyses by hypertension status revealed a negative association between NHHR and FT4-related ratios in both hypertensive and non-hypertensive groups. Subgroup analyses by diabetes, CVD, and BMI categories consistently showed that NHHR was negatively associated with FT4 and FT4/FT3 ratios. These findings suggest that NHHR may influence thyroid function differently across gender, age, and health conditions, with a general trend of negative associations with FT4 and FT4/FT3 across various subgroups.

### Sensitivity analyses

To ensure the robustness of our findings, we conducted sensitivity analyses. First, we excluded individuals diagnosed with hyperthyroidism, hypothyroidism, or thyroid cancer. We then applied weighted linear regression to evaluate the association between NHHR and thyroid hormone levels, and the results remained consistent and stable (**Supplementary Material 10**).

## Discussion

The results found that NHHR is negatively association with FT4, the FT4/FT3, and TT4/FT3. The distinct non-linear relationships between NHHR and FT3, TT3, TT4, and the FT4/TT4 ratio suggest that changes in NHHR may have a more complex impact on the regulation of these thyroid hormones. In particular, the presence of non-linear associations may indicate that at certain levels of NHHR, thyroid hormone regulation could exhibit a threshold effect. That is, once NHHR reaches a critical point, the secretion or conversion rate of thyroid hormones may undergo a dramatic shift, increasing the sensitivity of thyroid function or making it more prone to disruption. This phenomenon could be due to the differential effects of NHHR on various metabolic pathways, further revealing the complexity of the interactions between metabolism and hormone balance. NHHR is an extremely valuable tool in clinical practice, crucial for predicting thyroid hormone levels and function (29, 39). For instance, patients with metabolic syndrome and dyslipidemia are at high risk for thyroid disorders (39). As an effective indicator for assessing metabolic syndrome, NHHR may also play a significant role in the early detection of thyroid diseases (29). For patients who exhibit thyroid dysfunction, regular monitoring of NHHR values can help doctors adjust treatment plans and effectively control hormone level fluctuations (29, 40). This is particularly critical for promoting healthy growth in children (41). Additionally, research indicates that NHHR is an effective tool for assessing the risk of CVD (42). Both hypothyroid and hyperthyroid patients are at increased risk for CVD (7, 9), and patients undergoing cardiac surgery may also develop acquired hypothyroidism (43). Thus, utilizing NHHR can more effectively manage the progression and prognosis of thyroid-related diseases in clinical practice.

This study discovered a positive association between NHHR and both TT3 and FT3 while noting a negative association with FT4. Although no studies directly report these relationships, previous investigations into the interaction between thyroid hormones and lipid abnormalities lend support to our findings. For example, a study in Malaysia demonstrated a notable negative association between FT3 levels and HDL-C (44). Roos and colleagues observed that FT4 showed a negative association with LDL-C and TG yet exhibited a positive association with HDL-C (45). Likewise, studies involving the Spanish population revealed a positive association between FT4 levels and HDL-C, alongside a marked negative association with LDL-C (46). A different research study showed that TT3 has a negative association with LDL-C by increasing the gene expression of LDL-C receptors and enhancing the clearance rate of LDL-C (47). Additionally, elevated levels of HDL-C and reduced levels of LDL-C are associated with a heightened risk of CVD (48). Many researchers have noted that the ratio of FT3/FT4, utilized to evaluate the conversion rate from thyroxine TT4 to triiodothyronine TT3, associates with heightened mortality rates from CVD (49). Consequently, there exists a remarkable link between high HDL-C, low LDL-C, and heightened TT3 levels (48, 49). Moreover, research indicates that heightened levels of NHHR are connected to an amplified risk of type 2 diabetes in patients with hyperthyroidism (42). Leptin, present in the adipose tissue of type 2 diabetes patients (50), can elevate TSH levels, thereby boosting deiodinase activity, facilitating the conversion of FT4 to FT3, and augmenting basal metabolic rate (51). Thus, the escalation in NHHR closely correlates with the decrease in FT4 and the increase in FT3. An animal experiment on obese dogs revealed that adipose tissue within obese animals stimulates the conversion of TT4 to TT3 in order to increase energy expenditure; 42% of obese dogs showed a decrease in FT4 concentration (52, 53). During this process, the transcriptional regulation of genes in adipose tissue contributes to elevated TG levels and decreased HDL-C levels (54). These findings reflect the correlation between NHHR and thyroid hormone levels, aligning with our conclusions. This association emphasizes the importance of NHHR in detecting thyroid hormones.

The underlying mechanisms of this relationship may involve several aspects: First, thyroid hormones increase the flow of bile acids, which promotes the liver’s consumption and synthesis of cholesterol, as well as its uptake from the blood (55, 56). This helps maintain a cholesterol balance in the liver. However, elevated NHHR levels disrupt this balance, leading to increased conversion of stored TT4 into the active form TT3 to restore equilibrium (57). Second, leptin, an essential neuroendocrine regulator, modulates the hypothalamic-pituitary-thyroid axis and governs the thyrotropin-releasing hormone production in the paraventricular and arcuate nuclei (58). An increase in lipid content raises leptin levels, which may lower FT4 levels by influencing serum TSH levels (59). Studies indicate that while FT4 is negatively associated with BMI, TSH is positively associated (60, 61). Moreover, the higher TSH and TT3 levels seen in individuals with obesity might reflect a physiological adaptation aimed at enhancing energy expenditure and managing body weight (57). Third, cholesterol is vital for the production of thyroid hormones (62). High lipid levels are associated with thyroid dysfunction and metabolic disturbances, which can affect the synthesis of thyroid hormones, potentially promoting the synthesis of TT3, although the impact on TT4 synthesis and release may vary (63). Lastly, elevated non-HDL-C levels are often associated with a low-grade inflammatory state, which could affect hormone metabolism (64). Research shows that inflammatory factors can enhance the expression of deiodinases, enzymes that specifically remove iodine atoms from TT4, producing active TT3 and inactive diiodothyronine (65, 66). Thus, inflammation may promote the conversion of TT4 to TT3 while reducing the overall level of TT4.

## Strength and limitation

This research possesses multiple advantages. Firstly, it represents the most extensive population-based analysis conducted thus far to examine the associations of NHHR with various thyroid hormones. Secondly, our analysis utilized a sample that represents the entire U.S. adult demographic to depict the general population accurately. Additionally, we carried out a comprehensive analysis of different variables and made adjustments for covariates to confirm the reliability of our results.

Several limitations should be considered when interpreting the findings of this study. First, the cross-sectional design limits our ability to establish a causal relationship between NHHR and thyroid hormones. Although a potential association between these two factors was suggested in the present study, the simultaneous measurement of lipid biomarkers and thyroid hormone levels prevents us from determining the directionality or temporality of this relationship. Specifically, dyslipidemia, as indicated by an elevated NHHR, may impact thyroid function through mechanisms such as altered deiodination or disruption of thyroid hormone transport. Conversely, thyroid hormones have also been reported to play a crucial role in regulating lipid metabolism. Changes in thyroid function can lead to alterations in lipid profiles, including HDL-C and non-HDL-C cholesterol levels. The possibility of bidirectional interactions introduces the potential for reverse causality, where changes in thyroid hormones could influence NHHR rather than the other way around.

Secondly, despite adjusting for various confounding factors, residual confounding remains an issue that warrants attention. For example, during the NHANES 2007-2012 period, data on inflammatory markers were lacking. However, changes in inflammatory markers may concurrently affect both NHHR and thyroid hormone levels, thereby leading to biased results. Our analysis may not have sufficiently accounted for this potential factor.

Additionally, we conducted MR analysis to further investigate the relationship. Unfortunately, there is no available GWAS specifically focused on NHHR, and the GWAS summary data for TT3, FT3, TT4 and FT4 are also restricted (67). Therefore, we utilized GWAS data from the Global Lipids Genetics Consortium for HDL, LDL, and TC as exposures (32), and the UKB-based GWAS for hyperthyroidism and hypothyroidism as binary outcomes. However, no significant results were observed. In addition to the racial differences (mixed American vs. European descent), the discrepancy between the cross-sectional findings and MR results, we think, may be due to the use of related, but not fully equivalent, phenotypes. To further investigate, we analyzed the associations between HDL-C, LDL-C, TC, and these two binary phenotypes using NHANES data, and again, no significant results were found. As highlighted in our Introduction section, NHHR may provide greater value for disease prediction than traditional lipid markers, emphasizing the importance of further research into NHHR, including dedicated GWAS.

Additionally, thyroid hormone levels were measured using immunoassay methods, which are known to have lower accuracy, especially at low concentrations. Although NHANES employed high-quality laboratory techniques, the lack of LC-MS/MS validation might introduce measurement bias. Future studies incorporating LC-MS/MS validation could provide more accurate and reliable results.

Finally, the NHANES population primarily represents the U.S. population, and the findings may not be fully generalizable to other populations. Cultural, dietary, and genetic differences may influence the association between NHHR and thyroid hormones in other regions, necessitating validation in diverse cohorts.

## Conclusions

In summary, this study revealed that among U.S. adults, an increase in NHHR was significantly negatively associated with FT4, FT4/FT3, and TT4/TT3. However, no significant association was found between HDL-C, LDL-C, TC, and the risk of hyperthyroidism or hypothyroidism, highlighting the importance of monitoring NHHR and thyroid hormone levels in clinical practice.

## Data Availability

Publicly available datasets were analyzed in this study. This data can be found here: The data in the current study can be found on the website: https://www.cdc.gov/nchs/nhanes/.
