# Supplementary material for: Association between non-high-density lipoprotein cholesterol to high-density lipoprotein cholesterol ratio and serum thyroid function measures: Recent Findings from NHANES 2007–2012 and Mendelian randomization
Source: Front Endocrinol (Lausanne). 2025 Jan 24;16:1467254. doi: 10.3389/fendo.2025.1467254 (PMC11802436; doi:10.3389/fendo.2025.1467254)
Supplement: Supplementary file 1 [file Table1.docx]

**Supplementary Material 1. Detailed information on covariate.**

| **Covariate** | **Range** | **Grouping for adjustment** |
| --- | --- | --- |
| **Race** | **NA** | Mexican American |
|  |  | Non-Hispanic white |
|  |  | Non-Hispanic black |
|  |  | Other races |
|  |  |  |
| **Education level** | **NA** | Less Than 9th Grade |
|  |  | 9-11th Grade (Includes 12th grade with no diploma) |
|  |  | College Graduate or above |
|  |  | High School Grad/GED or Equivalent |
|  |  | Some college or AA degree |
|  |  |  |
| **Income level [1]** | **PIR: 0.00-5.00 ("5.00" means ≥ 5.00)** | low-income PIR: (0.00-1.00] |
|  |  | middle-income PIR: (1.00-4.00) |
|  |  | high-income PIR: [4.00-5.00] |
|  |  |  |
| **Smoking status** | **NA** | Yes |
|  |  | No |
|  |  |  |
| **Alcohol consumption [2]** | **NA** | Heavy: ≥3 drinks per day for females or ≥4 drinks per day for males |
|  |  | Moderate: 2-3 drinks per day for females or 3-4 drinks per day for males |
|  |  | Mild: 1 drink per day for females or 1-2 drinks per day for males |
|  |  | Former: Self-reported former drinker |
|  |  | Never: Self-reported never drinker |
|  |  |  |
| **Diabetes [3]** | **NA** | 1.doctor told you have diabetes |
|  |  | 2. glycohemoglobin HbA1c(%) >= 6.5 |
|  |  | 3. fasting glucose (mmol/l) >= 7.0 |
|  |  | 4. random blood glucose (mmol/l) >= 11.1 |
|  |  | 5.two-hour OGTT blood glucose (mmol/l) >= 11.1 |
|  |  | 6.Use of diabetes medication or insulin |
|  |  |  |
| **Hypertension [4]** | **NA** | 1. self-reported diagnosis of hypertension |
|  |  | 2. SBP≥140 mmHg and/or DBP≥90 mmHg |
|  |  | 3. the use of antihypertensive medication |
|  |  |  |
| **Marital Status** | **NA** | Divorced |
|  |  | Living with partner |
|  |  | Married |
|  |  | Never married |
|  |  | Separated |
|  |  | Widowed |

**Reference:**

[1] The role of hypertension in bone mineral density among males older than 50 years and postmenopausal females: evidence from the US National Health and Nutrition Examination Survey, 2005-2010 - PubMed. https://pubmed.ncbi.nlm.nih.gov/37397722/. Accessed 25 Oct 2023

[2] Rattan P, Penrice DD, Ahn JC, et al (2022) Inverse Association of Telomere Length With Liver Disease and Mortality in the US Population. Hepatol Commun 6:399–410. https://doi.org/10.1002/hep4.1803

[3] Serum selenium concentrations and risk of all-cause and heart disease mortality among individuals with type 2 diabetes - PubMed. https://pubmed.ncbi.nlm.nih.gov/34664061/. Accessed 25 Oct 2023

[4] Chen L, Zhang J, Zhou N, et al (2023) Association of different obesity patterns with hypertension in US male adults: a cross-sectional study. Sci Rep 13:10551. <https://doi.org/10.1038/s41598-023-37302-x>

**Supplementary material 2. The information on the concentration of both independent and dependent variables.**

|  | Mean ± SD | GM (95% CI) | Q25 | Q50 | Q75 |
| --- | --- | --- | --- | --- | --- |
| NHHR | 2.929 ± 1.408 | 2.65 (1.103 ,6.367) | 1.961 | 2.661 | 3.606 |
| FT3 (pg/mL) | 3.223 ± 0.641 | 3.191 (2.468 ,4.125) | 2.950 | 3.200 | 3.410 |
| FT4 (pmol/L) | 10.462 ± 2.271 | 10.266 (7.079 ,14.887) | 9.000 | 10.300 | 11.600 |
| TSH (mIU/I) | 2.139 ± 2.867 | 1.624 (0.365 ,7.226) | 1.107 | 1.656 | 2.550 |
| TT3 (ng/dL) | 115.308 ± 25.117 | 112.898 (75.672 ,168.437) | 100.000 | 113.000 | 127.000 |
| TT4 (ug/dL) | 8.048 ± 1.692 | 7.88 (5.267 ,11.789) | 6.900 | 7.900 | 9.000 |
| FT4/FT3 | 3.295 ± 0.763 | 3.217 (2.107 ,4.913) | 2.812 | 3.214 | 3.625 |
| TT4/TT3 | 0.072 ± 0.019 | 0.07 (0.044 ,0.112) | 0.060 | 0.069 | 0.080 |
| FT4/TT4 | 1.325 ± 0.242 | 1.303 (0.909 ,1.868) | 1.170 | 1.309 | 1.468 |
| FT3/TT3 | 0.029 ± 0.006 | 0.028 (0.02 ,0.039) | 0.026 | 0.028 | 0.031 |
| SD, standard deviation; GM, geometric mean; CI, confidence interval; Q, quantiles. | | | | | |

**Supplementary material 3 Association between HDL-C, LDL-C, and TC with Hyperthyroidism and Hypothyroidism in U.S. Adults**

|  | **β^a^ (95% CI), P-value** | |
| --- | --- | --- |
|  | **Hyperthyroidism** | **Hypothyroidism** |
| **HDL-C** |  |  |
| **Model 1** | 1.22 (0.74, 2.00) 0.43 | 0.99 (0.87, 1.14) 0.94 |
| **Model 2** | 0.86 (0.48, 1.55) 0.60 | 0.97 (0.85, 1.11) 0.67 |
| **Model 3** | 0.86 (0.38, 1.93) 0.48 | 0.96 (0.84, 1.10) 0.65 |
|  |  |  |
| **LDL-C** |  |  |
| **Model 1** | 0.82 (0.67, 1.01) 0.06 | 0.99 (0.95, 1.03) 0.54 |
| **Model 2** | 0.79 (0.65, 1.00) 0.05 | 0.98 (0.94, 1.02) 0.31 |
| **Model 3** | 0.81 (0.64, 1.03) 0.06 | 0.98 (0.94, 1.03) 0.38 |
|  |  |  |
| **TC** |  |  |
| **Model 1** | 0.82 (0.68, 0.99) 0.05 | 0.99 (0.95, 1.04) 0.70 |
| **Model 2** | 0.80 (0.65, 0.99) 0.05 | 0.98 (0.94, 1.03) 0.40 |
| **Model 3** | 0.80 (0.65, 1.00) 0.06 | 0.98 (0.94, 1.03) 0.46 |

**Notes**: Due to the non-linear relationships between NHHR and FT3, TT3, TT4, and FT4/TT4, their results are not displayed in the linear regression analysis;**β^a^**: effect size**; Model 1^b^**: no covariables were adjusted; **Model 2^c^:** adjusted for sex, age, and race; **Model 3^d^:** adjusted for age, gender, race, marital status, urine iodine concentration, moderate recreational activities, education level, PIR, BMI, smoking status, alcohol consumption, diabetes, hypertension, lipid-lowering medications and thyroid hormone medications

**Abbreviations**: **NHHR**, non-high-density lipoprotein cholesterol to high-density lipoprotein cholesterol ratio; **95% CI**, 95% confidence interval; **OR**, odds ratio; **FT4**, free thyroxine; **TT4**, total thyroxine; **FT3**, free triiodothyronine; **TT3**, total triiodothyronine; **TSH**, thyroid-stimulating hormone. The bold values indicate statistically significant differences.

**Supplementary material 4 Relationship between NHHR and thyroid hormones analyzed by gender subgroups.**

| **Thyroid hormones** | **Male** | |  | **Female** | |
| --- | --- | --- | --- | --- | --- |
|  | ***β* (95% CI)** | **p-value** |  | ***β* (95% CI)** | **p-value** |
| **FT4** | -0.11 (-0.26, 0.04) | 0.13 |  | -0.20 (-0.41, 0.01) | 0.06 |
| **TSH** | 0.09 (-0.07, 0.26) | 0.25 |  | 0.46 (-0.34, 1.27) | 0.24 |
| **FT4/FT3** | **-0.07 (-0.13, -0.02)** | **0.01** |  | **-0.13 (-0.20, -0.06)** | **<0.001** |
| **TT4/TT3** | 0.000 (-0.001, 0.000) | 0.97 |  | **-0.001 (-0.002, -0.001)** | **<0.001** |

**Notes**: β: effect size; adjusted for age, race, marital status, urine iodine concentration, moderate recreational activities, education level, PIR, BMI, smoking status, alcohol consumption, diabetes, hypertension, lipid-lowering medications and thyroid hormone medications

**Abbreviations**: **NHHR**, non-high-density lipoprotein cholesterol to high-density lipoprotein cholesterol ratio; **95% CI**, 95% confidence interval; **OR**, odds ratio; **FT4**, free thyroxine; **TT4**, total thyroxine; **FT3**, free triiodothyronine; **TT3**, total triiodothyronine; **TSH**, thyroid-stimulating hormone. The bold values indicate statistically significant differences.

**Supplementary material 5 Relationship between NHHR and thyroid hormones analyzed by age subgroups.**

| **Thyroid hormones** | **≥60** | |  | **＜60** | |
| --- | --- | --- | --- | --- | --- |
|  | ***β* (95% CI)** | **p-value** |  | ***β* (95% CI)** | **p-value** |
| **FT4** | -0.22 (-0.54, 0.10) | 0.16 |  | -0.06 (-0.22, 0.09) | 0.40 |
| **TSH** | -0.02 (-0.30, 0.26) | 0.89 |  | 0.30 (-0.18, 0.78) | 0.20 |
| **FT4/FT3** | **-0.13 (-0.24, -0.02)** | **0.03** |  | **-0.10 (-0.15, -0.05)** | **<0.001** |
| **TT4/TT3** | -0.001 (-0.002, 0.000) | 0.05 |  | 0.000 (-0.001, 0.000) | 0.331 |

**Notes**: β: effect size; adjusted for gender, race, marital status, urine iodine concentration, moderate recreational activities, education level, PIR, BMI, smoking status, alcohol consumption, diabetes, hypertension, lipid-lowering medications and thyroid hormone medications

**Abbreviations**: **NHHR**, non-high-density lipoprotein cholesterol to high-density lipoprotein cholesterol ratio; **95% CI**, 95% confidence interval; **OR**, odds ratio; **FT4**, free thyroxine; **TT4**, total thyroxine; **FT3**, free triiodothyronine; **TT3**, total triiodothyronine; **TSH**, thyroid-stimulating hormone. The bold values indicate statistically significant differences.

**Supplementary material 6 Relationship between NHHR and thyroid hormones analyzed by hypertension subgroups.**

| **Thyroid hormones** | **Hypertension** | |  | **Non-Hypertension** | |
| --- | --- | --- | --- | --- | --- |
|  | ***β* (95% CI)** | **p-value** |  | ***β* (95% CI)** | **p-value** |
| **FT4** | -0.13 (-0.33, 0.07) | 0.18 |  | -0.11 (-0.30, 0.09) | 0.27 |
| **TSH** | 0.06 (-0.13, 0.25) | 0.52 |  | 0.32 (-0.36, 0.99) | 0.34 |
| **FT4/FT3** | **-0.14 (-0.22, -0.06)** | **0.002** |  | **-0.09 (-0.14, -0.03)** | **0.003** |
| **TT4/TT3** | **-0.001 (-0.002, 0.000)** | **0.003** |  | -0.001 (-0.001, 0.000) | 0.156 |

**Notes**: β: effect size; adjusted for age, gender, race, marital status, urine iodine concentration, moderate recreational activities, education level, PIR, BMI, smoking status, alcohol consumption, diabetes, lipid-lowering medications and thyroid hormone medications

**Abbreviations**: **NHHR**, non-high-density lipoprotein cholesterol to high-density lipoprotein cholesterol ratio; **95% CI**, 95% confidence interval; **OR**, odds ratio; **FT4**, free thyroxine; **TT4**, total thyroxine; **FT3**, free triiodothyronine; **TT3**, total triiodothyronine; **TSH**, thyroid-stimulating hormone. The bold values indicate statistically significant differences.

**Supplementary material 7 Relationship between NHHR and thyroid hormones analyzed by diabetes subgroups.**

| **Thyroid hormones** | **Diabetes** | |  | **Non- Diabetes** | |
| --- | --- | --- | --- | --- | --- |
|  | ***β* (95% CI)** | **p-value** |  | ***β* (95% CI)** | **p-value** |
| **FT4** | -0.12 (-0.51,0.26) | 0.51 |  | -0.14 (-0.32, 0.03) | 0.10 |
| **TSH** | 0.028 (-0.03,0.09) | 0.35 |  | 0.214 (-0.083, 0.511) | 0.151 |
| **FT4/FT3** | -0.14 (-0.29, 0.00) | 0.05 |  | -0.11 (-0.16, -0.06) | <0.001 |
| **TT4/TT3** | -0.001 (-0.002,0.000) | 0.180 |  | -0.001 (-0.002,0.000) | **0.004** |

**Notes**: β: effect size; adjusted for age, gender, race, marital status, urine iodine concentration, moderate recreational activities, education level, PIR, BMI, smoking status, alcohol consumption, hypertension, lipid-lowering medications and thyroid hormone medications

**Abbreviations**: **NHHR**, non-high-density lipoprotein cholesterol to high-density lipoprotein cholesterol ratio; **95% CI**, 95% confidence interval; **OR**, odds ratio; **FT4**, free thyroxine; **TT4**, total thyroxine; **FT3**, free triiodothyronine; **TT3**, total triiodothyronine; **TSH**, thyroid-stimulating hormone. The bold values indicate statistically significant differences.

**Supplementary material 8 Relationship between NHHR and thyroid hormones analyzed by CVD subgroups.**

| **Thyroid hormones** | **CVD** | |  | **Non- CVD** | |
| --- | --- | --- | --- | --- | --- |
|  | ***β* (95% CI)** | **p-value** |  | ***β* (95% CI)** | **p-value** |
| **FT4** | -0.28 (-0.69, 0.14) | 0.17 |  | -0.12 (-0.26, 0.02) | 0.10 |
| **TSH** | -0.31 (-0.70, 0.08) | 0.11 |  | 0.28 (-0.17, 0.73) | 0.21 |
| **FT4/FT3** | -0.11 (-0.31, 0.09) | 0.25 |  | -0.11 (-0.16, -0.07) | <0.001 |
| **TT4/TT3** | -0.001 (-0.002, 0.000) | 0.003 |  | -0.001 (-0.001, 0.000) | 0.016 |

**Notes**: β: effect size; adjusted for age, gender, race, marital status, urine iodine concentration, moderate recreational activities, education level, PIR, BMI, smoking status, alcohol consumption, hypertension, lipid-lowering medications and thyroid hormone medications

**Abbreviations**: **NHHR**, non-high-density lipoprotein cholesterol to high-density lipoprotein cholesterol ratio; **95% CI**, 95% confidence interval; **OR**, odds ratio; **FT4**, free thyroxine; **TT4**, total thyroxine; **FT3**, free triiodothyronine; **TT3**, total triiodothyronine; **TSH**, thyroid-stimulating hormone. The bold values indicate statistically significant differences; **CVD**, cardiovascular disease

**Supplementary material 9 Relationship between NHHR and thyroid hormones analyzed by BMI subgroups.**

| **Thyroid hormones** | **BMI≥ 30 kg/m^2^** | |  | **BMI < 30 kg/m^2^** | |
| --- | --- | --- | --- | --- | --- |
|  | ***β* (95% CI)** | **p-value** |  | ***β* (95% CI)** | **p-value** |
| **FT4** | -0.26 (-0.52, 0.01) | 0.060 |  | -0.14 (-0.33, 0.04) | 0.13 |
| **TSH** | 0.15 (-0.09, 0.38) | 0.22 |  | 0.25 (-0.29, 0.80) | 0.34 |
| **FT4/FT3** | -0.12 (-0.20, -0.03) | 0.01 |  | -0.11 (-0.16, -0.05) | 0.001 |
| **TT4/TT3** | -0.001 (-0.002, 0.000) | 0.009 |  | -0.001 (-0.002, 0.000) | 0.011 |

**Notes**: β: effect size; adjusted for age, gender, race, marital status, urine iodine concentration, moderate recreational activities, education level, PIR, smoking status, alcohol consumption, hypertension, lipid-lowering medications and thyroid hormone medications

**Abbreviations**: **NHHR**, non-high-density lipoprotein cholesterol to high-density lipoprotein cholesterol ratio; **95% CI**, 95% confidence interval; **OR**, odds ratio; **FT4**, free thyroxine; **TT4**, total thyroxine; **FT3**, free triiodothyronine; **TT3**, total triiodothyronine; **TSH**, thyroid-stimulating hormone. The bold values indicate statistically significant differences.

**Supplemental Material 10 Association between NHHR and serum thyroid hormones in U.S. adults after excluding special populations.**

| **serum thyroid hormones** | **β^a^ (95% CI), P-value** | | |
| --- | --- | --- | --- |
|  | **Model 1^b^** | **Model 2^c^** | **Model 3^d^** |
| **FT4** | -0.06 (-0.13, 0.00) 0.06 | **-0.08 (-0.15, -0.01) 0.02** | **-0.09 (-0.16, -0.02) 0.01** |
| **TSH** | 0.10 (-0.05, 0.24) 0.18 | 0.14 (-0.04, 0.33) 0.13 | 0.15 (-0.06, 0.36) 0.15 |
| **FT4/FT3** | **-0.07 (-0.09, -0.05) <0.001** | **-0.06 (-0.08, -0.04) <0.001** | **-0.06 (-0.08, -0.04) <0.001** |
| **TT4/TT3** | **-0.001 (-0.002, -0.001)** **<0.001** | **-0.001 (-0.001,0.000) 0.01** | **-0.001 (-0.001,0.000) 0.005** |

**Notes**: β: effect size; adjusted for age, gender, race, marital status, urine iodine concentration, moderate recreational activities, education level, PIR, BMI, smoking status, alcohol consumption, diabetes, hypertension, lipid-lowering medications and thyroid hormone medications.

**Abbreviations**: **NHHR**, non-high-density lipoprotein cholesterol to high-density lipoprotein cholesterol ratio; **95% CI**, 95% confidence interval; **OR**, odds ratio; **FT4**, free thyroxine; **TT4**, total thyroxine; **FT3**, free triiodothyronine; **TT3**, total triiodothyronine; **TSH**, thyroid-stimulating hormone. The bold values indicate statistically significant differences.
